# Diagnosis of suspected pediatric distal forearm fractures with point-of-care-ultrasound (POCUS) by pediatric orthopedic surgeons after minimal training

**DOI:** 10.1186/s12880-024-01433-y

**Published:** 2024-09-27

**Authors:** Josephine Edith Pohl, Philipp Schwerk, René Mauer, Gabriele Hahn, Ricardo Beck, Guido Fitze, Jurek Schultz

**Affiliations:** 1 Department of Pediatric Surgery, University of Technology Dresden, Dresden, Saxony, Germany; 2 Department of Radiology, University of Technology Dresden, Dresden, Saxony Germany; 3 Faculty of Medicine Carl Gustav Carus, Institute for Medical Informatics and Biometry (IMB), University of Technology Dresden, Dresden, Saxony Germany

**Keywords:** Wrist-POCUS, Fracture Ultrasound, Distal Forearm Fractures, Ultrasonography, Human distal forearm fractures, Child, Ultrasound, X-rays, Retrospective study

## Abstract

**Background:**

Several studies have advocated the use of ultrasound to diagnose distal forearm fractures in children. However, there is limited data on the diagnostic accuracy of ultrasound for distal forearm fractures when conducted by pediatric surgeons or trainees who manage orthopedic injuries in children. The objective of this study was to determine the diagnostic accuracy of point-of-care ultrasound (POCUS) for pediatric distal forearm fractures when conducted by pediatric surgeons and trainees after minimal training.

**Methods:**

This diagnostic study was conducted in a tertiary hospital emergency department in Germany. Participants were children and adolescents under 15 years of age who presented to the emergency department with an acute, suspected, isolated distal forearm fracture requiring imaging. Pediatric surgeons and trainees, after minimal training for sonographic fracture diagnosis, performed 6-view distal forearm POCUS on each participant prior to X-ray imaging. All data was retrospectively collected from the hospital’s routine digital patient files. The primary outcome was the diagnostic accuracy of POCUS compared to X-ray as the reference standard.

**Results:**

From February to June 2021, 146 children under 15 met all inclusion and exclusion criteria, and 106 data sets were available for analysis. Regarding the presence of a fracture, X-ray and Wrist-POCUS showed the same result in 99.1%, with 83/106 (78.3%) fractures detected in both modalities and one suspected buckle fracture on POCUS not confirmed in the radiographs. Wrist-POCUS had a sensitivity of 100% (95% CI [0.956, 1]) and a specificity of 95.8% (95% CI [0.789, 0.999]) compared to radiographs. In 6 cases, there were minor differences regarding a concomitant ulnar buckle. The amount of prior ultrasound training had no influence on the accuracy of Wrist-POCUS for diagnosing distal forearm fractures. All fractures were reliably diagnosed even when captured POCUS images did not meet all quality criteria.

**Conclusion:**

Pediatric surgeons and trainees, after minimal training in POCUS, had excellent diagnostic accuracy for distal forearm fractures in children and adolescents using POCUS compared to X-ray.

## Introduction

The most common fractures (29%) in children aged between 0 and 15 years are distal forearm fractures [1, 2]. Among these are buckle fractures resulting from metaphyseal compression without cortical disruption and metaphyseal fractures of different degrees with or without involvement of the physis [1–3]. The frequency of these fractures underlines the importance of a robust diagnostic pathway that reduces radiation to as low as reasonably achievable (ALARA). Ultrasound has been described as an alternative diagnostic method that reliably detects distal forearm fractures in children [4–12]. Compared to X-rays, point-of-care wrist ultrasound (Wrist-POCUS) is an imaging method that spares radiation [8]. Also, various studies have shown that Wrist-POCUS is quick and easily done [5, 10]. Furthermore, children do not need to be separated from their parents during Wrist-POCUS for radiation protection. At the same time, Wrist-POCUS might be less painful, as it is unnecessary to orient the arm to the X-ray machine [9–11].

Recommendations on performing POCUS in suspected distal forearm fractures have been around for decades [4–6, 13]. The ultrasound in six distinct planes, together with a diagnostic flow chart, as proposed by Ackermann [5, 14], was adopted by the German Society for Ultrasound in Medicine (DEGUM) [15]. Nevertheless, Wrist-POCUS is still not used regularly as the standard method in many centers. For one, the "[i]mplementation of new diagnostic imaging in existing health systems can expect resistance on different levels" [7]. Moreover, "[c]onventional x-ray is widely used, stored and easily retrieved by all personnel" [7]. A recent review identified only two out of nine studies on ultrasound in diagnosing distal forearm fractures to include orthopedic surgeons and argues that "ultrasound has not been proven as a suitable substitute […] in the detection of pediatric distal radius fractures" [16]. Therefore, we assessed the accuracy of Wrist-POCUS for diagnosing distal forearm fractures, performed by pediatric surgeons and pediatricians of differing training levels regardless of their formal ultrasound training. Furthermore, we calculated its hypothetical radiation-sparing effect in our cohort. We proposed a standardized operating pathway (SOP) for diagnosing distal forearm fractures primarily with ultrasound and standard radiographs only when needed in selected cases.

## Methods

### Study design, setting, and participants

From February to June 2021, all pediatric surgeons diagnosing and treating fractures in our tertiary center’s Emergency Department (ED) were asked to perform POCUS of the distal forearm in all acutely presenting cases of suspected, isolated distal forearm fractures in children up to 14 years after adequate trauma (e.g., falling on the outstretched hand, direct impact by a soccer ball, or other accidents) before sending for X-rays. Tenderness or swelling of the distal forearm supported the tentative diagnosis. Patients with apparent angulation, open fracture, polytrauma, and diagnoses that would affect bone development, such as osteogenesis imperfecta, were excluded. Starting in July 2022, all data was retrospectively collected from the hospital software and analyzed by the authors of this study. The Ethical Board of our university approved this study under approval EK 433102016. Patients or the public were not involved in our research's design, conduct, reporting, or dissemination plans. We asked parents and all children who could understand and answer for their consent, regardless of age.

### Description of scanners

In our ED, children younger than 15 are exclusively treated by pediatric surgeons, trainees in pediatric surgery, or pediatricians in training while on pediatric surgery rotation. All pediatric surgeons/trainees who worked in the ED to diagnose suspected distal forearm fractures completed at least three exams, with a maximum of 20 (Table 1). Only one examiner had formal bone ultrasound training before this study (Table 1).

**Table 1:** Qualification and Ultrasound Training of Involved Pediatric Surgeons and the Number of Examinations they performed

Position in Pediatric Surgery (except Trainee 9, who trained to become a pediatrician)	General ultrasound training including abdomen, brain, and urinary tract	Formal fracture ultrasound training	Number of exams performed
Trainee 1 (5th year training)	Yes	No	14
Trainee 2 (1st year training)	No	No	4
Trainee 3 (final year training)	Yes	No	4
Consultant 1 (1st year consultant)	Yes	No	11
Trainee 4 (4th year training)	Yes	No	7
Trainee 5 (4th year training)	Yes	No	17
Trainee 6 (3rd year training)	Yes	No	12
Trainee 7 (1st year training)	No	No	20
Trainee 8 (5th year training)	Yes	No	3
Consultant 2 (3rd year consultant)	Yes	Yes	10
Trainee 9 (3rd year pediatric training)	No	No	4

### Study procedures

**Fig. 1:**
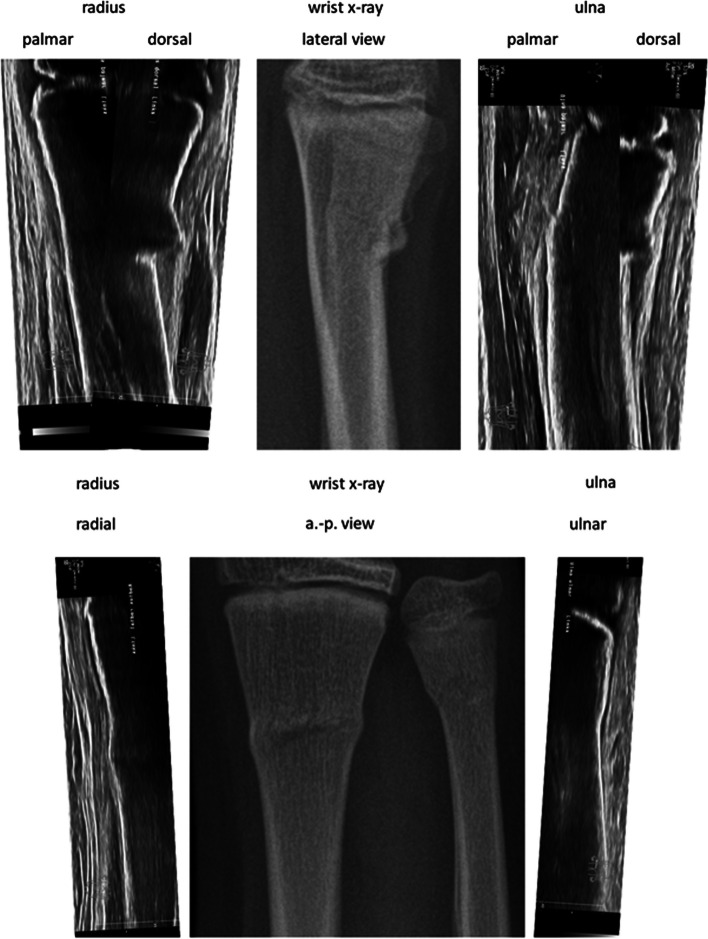
Merged, cut, and sized Wrist-POCUS of distal forearm buckle with corresponding X-rays for comparison

**Fig. 2:**
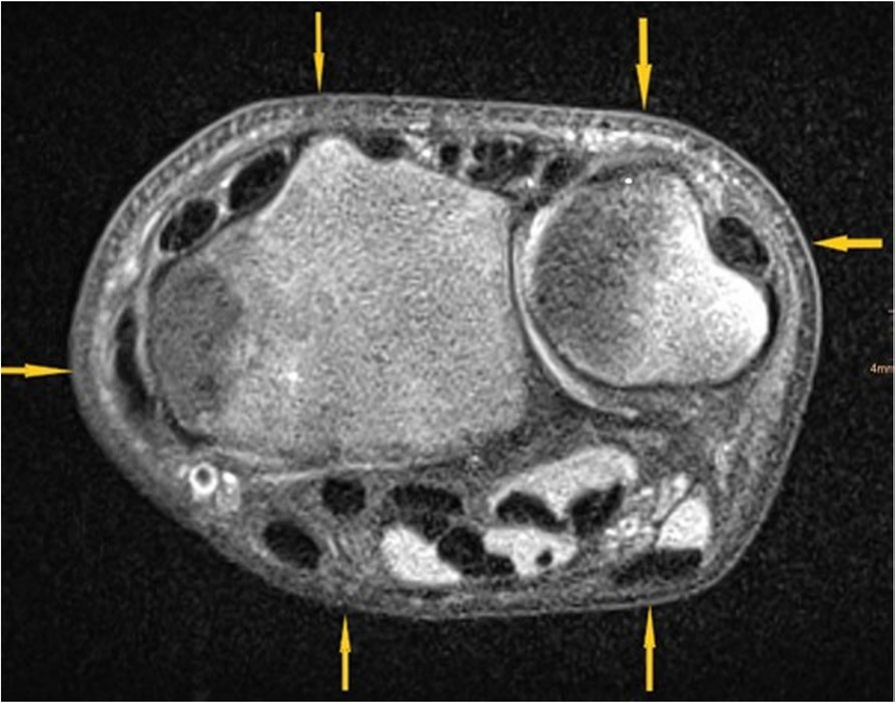
Six Wrist-POCUS planes illustrated in a distal forearm axial MRI-Scan

After collecting the medical history and the physical examination, the treating pediatric surgeon (Table 1) performed Wrist-POCUS directly in the ED using a linear transducer on one of the following standard ultrasound machines: Affinity 70, L18-5 (Koninklijke Philips N.V., Amsterdam, Netherlands) or Z.One, L14-5w (Zonare Medical Systems GmbH, Erlangen, Germany). An example of all 6 POCUS-views merged with the traditional X-ray can be seen in Fig. 1. Images were stored and retrieved from our Picture Archiving and Communication System (PACS). The integration of POCUS images was initially accomplished via the standard multi-step protocol used for radiographs. Later, a simplified protocol was established, facilitating the transfer of POCUS images to PACS. The radius and ulna of the affected forearm were depicted in six longitudinal sections: radius dorsal, lateral, and palmar, as well as ulna palmar, medial, and dorsal (Fig. 2). Wrist-POCUS was evaluated by the surgeon directly after performing the wrist POCUS according to previously published criteria: A fracture was diagnosed in case of a cortical gap, a kink, a torus formation, or a displacement [5]. Only after documenting the result of the Wrist-POCUS independent of PACS was the patient sent to radiology. The pediatric surgeon then evaluated a standard X-ray in two planes (anterior–posterior and lateral view). The reported result of the Wrist-POCUS and the X-ray images were then used to determine the therapeutic process. Subsequently, the X-ray image was evaluated independently by a pediatric radiologist blinded to the surgeon’s reports.

### Interventions

In December 2020, a senior pediatric surgeon formally trained in Wrist-POCUS held a 30-min in-house talk to all physicians working in the Department of Pediatric Surgery. In this talk, he demonstrated the theory of bone ultrasound, the six sonographic planes of the distal forearm, their quality criteria, the relevant ultrasound machine settings, and what to expect on Wrist-POCUS in various fractures. Additionally, all our pediatric surgeons were made familiar with the recommendation for Wrist-POCUS documentation by the German Society for Sonography in Medicine (DEGUM) [15]. No Wrist-POCUS had been documented before December 2020 in our clinic.

### Outcome measures

For the sensitivity and specificity calculation, only the diagnosis of any radius fracture was analyzed since detected simultaneous distal ulna fractures (buckle or fractured styloid process) would not have changed the clinical management, and no isolated ulna fractures were found. This is consistent with previous studies on this subject [7]. When the POCUS was initially reported as a “suspected” buckle, we counted this index-test result as “inconclusive.” As secondary outcome measures, we quantitatively assessed the training and experience of physicians who performed the index test. Additionally, we evaluated all Wrist-POCUS examinations according to the following criteria: 1) The six standard planes of Wrist-POCUS are captured. 2) The distal edge of the distal epiphysis represents the beginning of the image Sect. 3) The image is orthogonal and parallel to the bone over the entire scan length, ensuring crisp imaging of the soft tissue-cortical interface. 4) There is clear marking of sides and projections for good reproducibility. Finally, we evaluated the individual radiation doses of all X-rays analyzed for this study. When Wrist-POCUS was sufficient for the diagnosis, and no additional relevant information was gained by the subsequent X-rays, we deemed these X-rays as “avoidable.” Based on this evaluation, we calculated the radiation dose that could potentially be spared using Wrist-POCUS according to our proposed SOP.

### Data retrieval and analysis

JS (senior consultant in pediatric surgery), JP (researcher trained in the evaluation of POCUS), and PS (senior consultant in pediatric surgery) reviewed all five data sources after their retrieval, collection, and extraction from the hospital’s computer systems: the POCUS images, X-ray images, and the surgeons' reports on the POCUS and the X-ray, as well as the radiologists' reports. Radiographs and radiologists’ initial reports were reviewed for this study by GH (Head of pediatric radiology). GF (Head of pediatric surgery) was included to resolve discrepancies if needed. Sensitivity, specificity, Cohen's kappa, and their corresponding 95% confidence intervals (95% CI) were calculated.

## Results

**Fig. 3:**
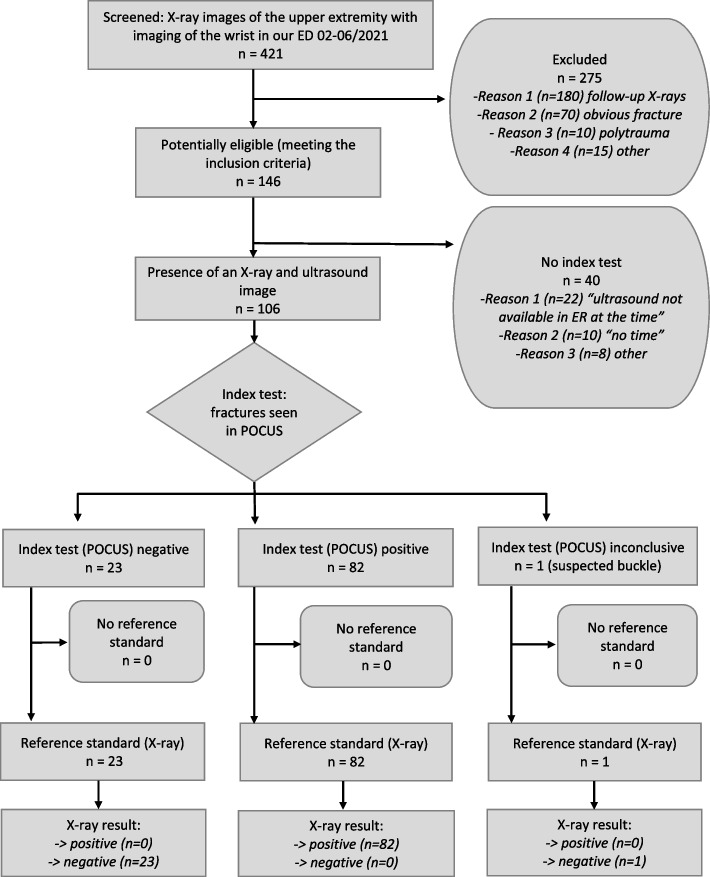
Patient Flow Diagram according to STARD 2015 guidelines [17]

A total of 3651 children and teenagers presented to the ED of our University Hospital during the analyzed 5-month period from February 2021 to June 2021, and 421 patients under 15 received an X-ray of the upper extremity with imaging of the distal forearm or wrist. These 421 patients were screened, and 146 patients met all inclusion and exclusion criteria; in 106 cases, complete data sets with POCUS report and subsequent X-rays, initial X-ray report, and final radiologist’s report on those X-rays were available (Fig. 3). This study population (*N* = 106) comprised 54 females and 52 males.The mean age was 9.29 (3 to 14 years). Wrist-POCUS was performed by 11 different physicians (8 surgical trainees, one pediatrician in training on surgical rotation, and two consultants) who detected fractures in 83 of 106 cases (78.3%). In one case, this diagnosis was suspected in Wrist-POCUS but was not seen on the X-ray.

Table 2 shows the number and distribution of 99 cases in which radiography and Wrist-POCUS fully agreed. "Other distal radial fractures," which include Aitken and greenstick fractures with or without ulna involvement, are listed separately because these fractures sometimes require reduction, external retention, or internal fixation. On the other hand, buckle fractures are only treated for pain relief.

**Table 2:** Number and distribution of cases in which radiography and Wrist-POCUS agreed

n	X-ray	Wrist-POCUS
23	no fracture	no fracture
56	buckle fracture of the radius	buckle fracture of the radius
20	other distal radial fracture	radial fracture

In addition to the index question (fracture present or not), Table 3 details seven cases with minor inconsistencies. In one case, a radial buckle fracture was suspected on Wrist-POCUS but was not confirmed on X-rays. In seven other cases, there were differences in the diagnosis of the ulna and ulnar styloid processus involvement, as seen in Table 3. Considering fracture detection, Wrist-POCUS had a sensitivity of 100% (95% CI [0.956,1]) and a specificity of 95.8% (95% CI [0.789,0.999]), compared with X-ray. X-ray and Wrist-POCUS showed the same result in 99.1% (Table 4). Cohen's kappa was 0.973 (95% CI [0.919, 1]), corresponding to a complete agreement. There were no reported adverse events, e.g., need for sedation, arterial hypotension, unusual pain, etc., during POCUS or X-rays.

**Table 3:** Inconsistent Findings

n	X-ray	Wrist-POCUS
1	no fracture	suspected radial buckle fracture
1	radial buckle fracture	buckle fracture radial + ulnar
3	buckle fracture radial + ulnar	radial buckle fracture
2	radial buckle fracture + ulna styloid fracture	radial buckle fracture

**Table 4:** Contingency table

		X-ray		
		Fracture	No Fracture	**Total**
POCUS	Fracture	82	1	83
	No Fracture	0	23	23
	**Total**	82	24	106

Apart from the seven minor inconsistencies between the final radiographic results and POCUS, we found 14 inconsistencies when comparing the initial surgeon’s and radiographer’s reports on the X-rays. These were about the simultaneous involvement of the ulna in 12 patients. However, next to the discrepancies concerning the ulna involvement, we found one discrete radial buckle and one non-displaced Aitken 1 fracture of the distal radius that were initially missed on the X-rays.

In 102 of 106 patients, we could retrieve the original POCUS images and review them for image quality criteria. In 92% (94/102) of the cases, six ultrasound planes were depicted, and complete labeling was available. Clear visualization of the bone by orthogonal and parallel scan was done in 79% (472/596) of all images. The distal edge of the epiphysis represented the beginning of the image in 49% (292/596) of all images. In all 102 evaluated cases, the surgeon’s initial judgment concerning the POCUS was agreed upon during the retrospective review process. The average total area dose product applied to acquire the evaluated X-rays was 0.7µGy*m^2^. Following our new SOP, 81% of the included patients could have been diagnosed without X-rays. This could have potentially spared 44µGy*m^2^ in total.

## Discussion

With a sensitivity of 100% and a specificity of 95.8%, Wrist-POCUS had excellent diagnostic accuracy in this study. One radial buckle fracture was suspected on ultrasound but not reported on X-rays. All other discrepancies were due to the additional involvement of the ulna in radial buckle fractures, which did not affect treatment. When most studies have reported ultrasound examinations performed by radiologists, emergency physicians, or trained orthopedic surgeons [7], we found equally good results when pediatric residents, surgical residents, and consultants performed ultrasound exams after only 30 min of theoretical education. Intriguingly, fractures were reliably diagnosed even when many saved ultrasound images did not meet all quality criteria. This study should encourage using Wrist-POCUS to diagnose suspected forearm fractures in children and adolescents in most settings, especially if conventional X-rays are available in case of remaining doubts after POCUS.

Wrist-POCUS has a critical advantage since it reduces radiation exposure compared with the standard diagnostic modality of forearm X-rays. This is particularly important as children are more susceptible to radiation due to hematopoietic marrow in the extremities, higher mitosis rates, and longer remaining life expectancies [18]. While pediatric radiologists were practicing radiation protection by the principles of ALARA, the average total area dose product for the children examined was still 0.7µGy*m^2^. Although already a very low dose, this dose should still be avoided, if possible [19]. After all, in 81% of our patients presenting with post-traumatic forearm pain, radiation could have been completely avoided, thus potentially sparing 44µGy*m^2^ in total.

Previous publications have demonstrated that children reported less pain during Wrist-POCUS; moreover, parents also stated their children tolerated ultrasound better than X-rays [4, 10, 20]. In addition to the advantages of Wrist-POCUS previously published, and despite ultrasound usually being performed by the treating physician, it may also save time. Compared to an X-ray, Wrist-POCUS does not necessitate transferring the patient to the X-ray department or waiting for the examination and radiological evaluation [4, 5]. One physician can perform all management aspects, possibly resulting in a closer patient-physician relationship and economic advantages [5, 11, 13]. This study shows that ultrasound images can be stored, archived, retrieved, and reviewed with any standard Picture Archiving and Communication System, just like radiographs. Finally, Germany's health- and accident insurances pay identical tariffs for bone ultrasound and conventional radiographs in two planes.

**Fig. 4:**
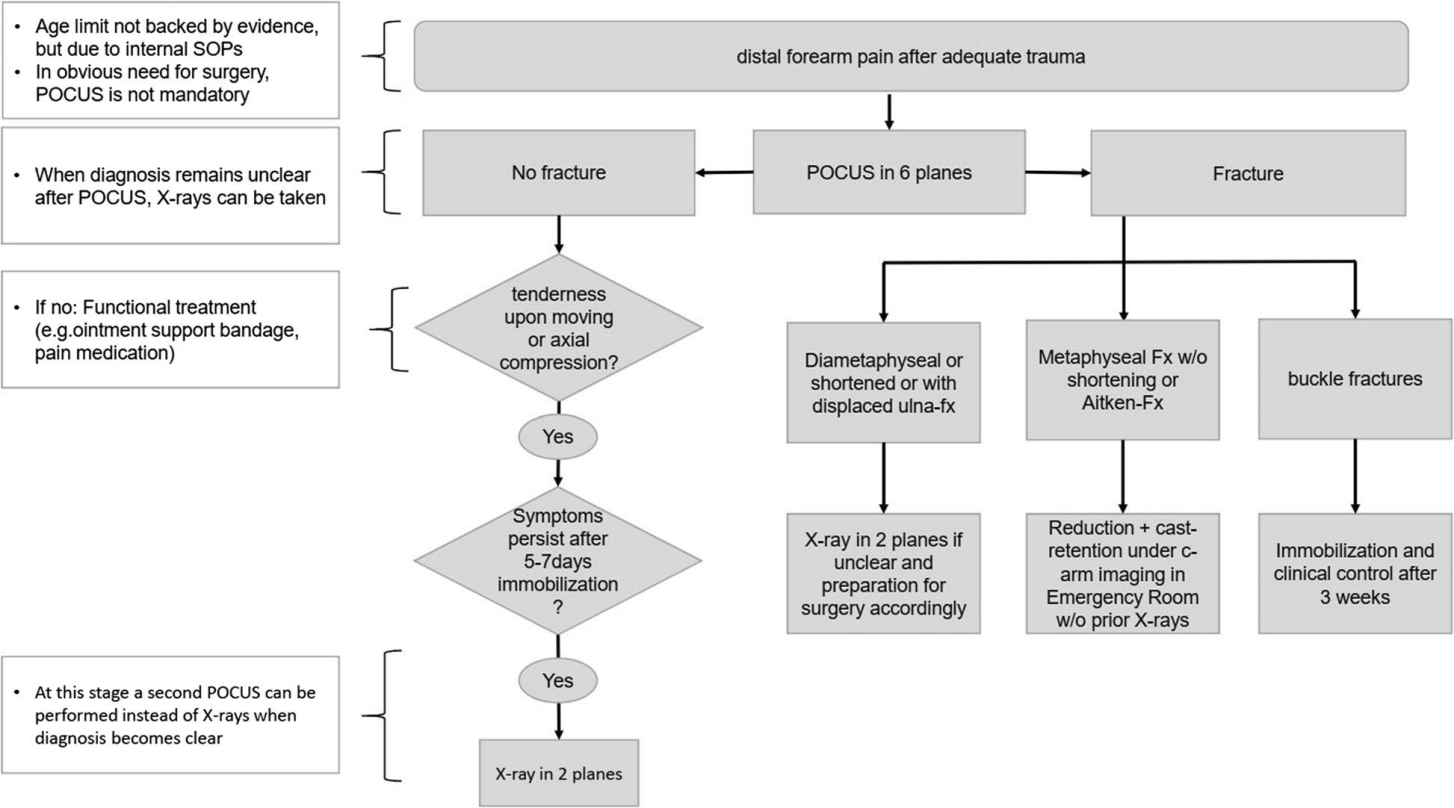
Standard Operating Procedure established as a result of this study's outcomes

In most cases, treatment of distal forearm fractures requires only conservative care [21, 22]. Many of these fractures could be treated in settings without access to X-ray facilities or radiologists. Future studies might demonstrate that ultrasound can partly replace X-ray imaging even in follow-up examinations or to evaluate reductions [23]. Nevertheless, the aim is to establish Wrist-POCUS as the first-choice tool for detecting distal forearm fractures. Therefore, we adapted the "Wrist-Safe" [14] to our ED’s SOP (Fig. 4): Whenever a child or adolescent presents with distal forearm pain after adequate trauma, we initially perform Wrist-POCUS in six planes. Wrist-POCUS is not mandatory in case of an obvious need for reduction or surgery. If no fracture is detected via Wrist-POCUS, but severe pain when moving or upon axial compression is present, we recommend immobilization for 5–7 days as a pain treatment. In cases of persisting symptoms, Wrist-POCUS is to be repeated for possible detection of a missed fracture or callus [24]. A two-plane X-ray is mandatory if a fracture can still not be detected. Once Wrist-POCUS confirms a fracture, there are three different ways of proceeding depending on the fracture type: In case of a buckle fracture, the examination by Wrist-POCUS is sufficient. The fracture can be immobilized for pain relief and checked clinically after 2–3 weeks. In metaphyseal fractures with or without shortening or when diagnosing a dislocated epiphyseal fracture, reduction and cast retention are performed in ED under c-arm imaging. An X-ray may be taken beforehand if the treating surgeon doubts the need for fracture reduction after Wrist-POCUS. For diametaphyseal fractures or concomitant displaced ulna fractures, an X-ray in 2 planes in preparation for surgery is required. In general, performing an X-ray is always possible in case of any uncertainty regarding a fracture after Wrist-POCUS.

Limitations: Our study used X-rays as a reference standard, knowing that radiographs might also miss subtle fractures. More precisely, the index test consists of Wrist-POCUS, medical history, and clinical examination. However, the interpretation of radiographs was also influenced by medical history and clinical exam facts. Of 146 eligible patients, 40 were excluded as no Wrist-POCUS results were found. Technical difficulties and time efficiency in the emergency department were the main reasons for missing POCUS. These problems were later addressed with an additional ultrasound machine reserved for POCUS that is now integrated into our PACS via a quick protocol. Still, bias in selecting patients for Wrist-POCUS and a potential data recall bias cannot be excluded. The small sample size, monocentric, retrospective study design, and lack of imaging follow-up are more limitations. Despite these limitations, the retrospective evaluation of establishing Wrist-POCUS provides valuable insights into real-life clinical routines. Finally, for the calculation of diagnostic accuracy, there was no distinction made between buckle fractures and different cortical breach fractures. However, all radial buckles were correctly diagnosed with POCUS. All cortical breach fractures diagnosed on radiographs were also diagnosed with POCUS. More studies are needed to show the value of POCUS in guiding the management of different cortical breach fractures. Of course, our proposed SOP needs to be validated in future prospective studies.

Conclusion: In summary, this study shows that detecting distal forearm fractures with ultrasound can have excellent sensitivity and specificity even when not performed by specially trained sonographers but by pediatric surgeons and pediatricians of different training levels with little ultrasound training and no extensive training in Wrist-POCUS.

## Data Availability

All anonymized primary data is available from the corresponding author upon reasonable request.

## Abbreviations

ALARA: As low as reasonably achievable
DEGUM: German Society for Ultrasound in Medicine
ED: Emergency Department
PACS: Picture Archiving and Communication System
POCUS: Point-of-care-ultrasound
Wrist-POCUS: Point-of-care wrist ultrasound
